# At Least I Tried: The Relationship between Regulatory Focus and Regret Following Action vs. Inaction

**DOI:** 10.3389/fpsyg.2016.01684

**Published:** 2016-10-27

**Authors:** Adi Itzkin, Dina Van Dijk, Ofer H. Azar

**Affiliations:** Guilford Glazer Faculty of Business and Management, Ben-Gurion University of the NegevBeer Sheva, Israel

**Keywords:** regulatory focus, regulatory fit, promotion focus, prevention focus, regret, action, inaction

## Abstract

Regret is an unpleasant feeling that may arise following decisions that ended poorly, and may affect the decision-maker's well-being and future decision making. Some studies show that a decision to act leads to greater regret than a decision not to act when both resulted in failure, because the latter is usually the norm. In some cases, when the norm is to act, this pattern is reversed. We suggest that the decision maker's regulatory focus, affects regret after action or inaction. Specifically, promotion-focused individuals, who tend to be more proactive, view action as more normal than prevention-focused individuals, and therefore experience regulatory fit when an action decision is made. Hence, we hypothesized that promotion-focused individuals will feel less regret than prevention-focused individuals when a decision to act ended poorly. In addition, we hypothesized that a trigger for change implied in the situation, decreases the level of regret following action. We tested our hypotheses on a sample of 330 participants enrolled in an online survey. The participants received six decision scenarios, in which they were asked to evaluate the level of regret following action and inaction. Individual regulatory focus was measured by two different scales. Promotion-focused individuals attributed less regret than prevention-focused individuals to action decisions. Regret following inaction was not affected by regulatory focus. In addition, a trigger for change decreases regret following action. Orthodox people tend to attribute more regret than non-orthodox to a person who made an action decision. The results contribute to the literature by showing that not only the situation but also the decision maker's orientation affects the regret after action vs. inaction.

## Introduction

Every decision that we make in our life carries the risk that we might regret it. But what type of decisions will be regretted more: decisions of doing something or decisions of not doing anything? In the current paper we suggest that individual differences in regulatory focus would affect individuals' tendency to regret more what they did or what they did not do.

Regret is an unpleasant feeling that is aroused after retrospection that involves awareness to the negative aspects of a decision. The regret process involves running a mental re-creation of what actually happened vs. what could have happened, comparing these two options and deciding that the decision process and the outcome were suboptimal (Zeelenberg et al., 2002; Roese et al., 2009; Das and Joffe, 2012). The level of regret depends on an individual's perception of the mental gap between what happened as opposed to what could have happened. The greater the gap, the stronger the regret (Das and Kerr, 2010). Regret could lead to regret aversion, which further encourages people to learn from past decisions in order to avoid similar experiences in the future (Zeelenberg and Beattie, 1997; Zeelenberg et al., 2002; Roese et al., 2009; Das and Kerr, 2010; Das and Joffe, 2012).

There are contradictory findings regarding what induces more regret: action or inaction. Action is considered as doing something that changes the current situation, such as deciding to go out for dinner or changing a strategy when trying to solve a problem. Inaction, on the other hand, is considered as doing nothing or keeping the status quo, such as staying home or keeping the same strategy already used. Early research found that because inaction is usually the norm, action, which violates the norm, leads to greater regret (Kahneman and Tversky, 1982; Kahneman and Miller, 1986). This finding has been later replicated in numerous studies (e.g., Landman, 1987; Gleicher et al., 1990; Baron and Ritov, 1994; Gilovich and Medvec, 1995; Miller and Taylor, 1995; Ritov and Baron, 1995; N'gbala and Branscombe, 1997; Van der Pligt et al., 1998; Ordóñez and Connolly, 2000). This effect has been termed the *action effect*, namely, an action that leads to a failure will cause greater regret than inaction that leads to similar failure (Zeelenberg et al., 2002).

Yet, other studies demonstrated that under certain conditions inaction can produce more regret than action (Gilovich and Medvec, 1995; Zeelenberg et al., 2002). For example, Gilovich and Medvec (1995) indicated that regret perception depends on the time horizon. Specifically, action is more regrettable in the short term, while inaction is more regrettable in the long term. Following this view, Zeelenberg et al. (2002) broadened Kahneman and Tversky (1982) theory and have added the *inaction effect*, which becomes relevant when action is perceived as desirable and needed, whereas inaction is perceived as less desired. Zeelenberg et al. suggested that what is considered normal can be influenced by a relevant past decision. In particular, when prior outcomes are positive or absent, inaction is considered more normal and people will attribute more regret to action than to inaction. However, when prior outcomes are negative, action becomes the more normal decision and more regret will be attributed to inaction.

Bar-Eli et al. (2007) showed that sometimes action can be the norm even without prior outcomes. They found that soccer goalkeepers in penalty kicks perceive action (jumping to one of the sides) as more normal than inaction (staying at the center of the goal) and consequently failed inaction produces more regret than failed action. Consequently, goalkeepers almost always choose action even though this actually reduces their chances to stop the ball. Azar (2013) examined the impact of previous outcomes not on regret but on decisions in a business strategy context. He found that whether a strategy was previously successful or not did not affect the likelihood that it will be continued or changed, in scenarios where the previous outcome was not informative about the future, but could trigger emotional reaction, such as regret.

Other factors that affect the relationship between failure associated with action vs. inaction and level of regret are desirability and consistency (Seta et al., 2001). Seta et al. suggested that errors associated with action or inaction are less desirable and produce more regret when they are inconsistent with the decision maker's orientation (action or inaction) than when they are consistent. The effect of consistency and desirability was further demonstrated by McElroy and Dowd (2007). Building upon Seta et al. (2001) and McElroy and Dowd (2007) we suggest in the current paper that the level of regret following action or inaction is determined by the individual's regulatory focus, through the mechanism of regulatory fit. We rely on a well-established comprehensive motivational theory of regulatory focus (Higgins, 1997, 1998) to explain mixed evidences regarding regret following decisions of action vs. inaction.

Regulatory Focus Theory (Higgins, 1997, 1998), proposes that human motivation consists of two regulatory foci: promotion and prevention focus. People with promotion focus are motivated to achieve accomplishments, aspirations, and ideals, they are sensitive to gain - non-gain situations and to the presence or absence of positive outcomes. In contrast, individuals with prevention focus are motivated to attain security, responsibility, and duties. They are sensitive to loss - non-loss situations and to the presence or absence of negative outcomes (Higgins and Tykocinski, 1992; Friedman and Förster, 2001; Cesario et al., 2004; Förster et al., 2004). Regulatory focus can emerge as a chronic characteristic (personal disposition) as well as a situational (context-induced) variable (Higgins, 1997, 1998).

The two motivational foci are related to different types of strategies that are used to achieve individuals' goals. As was shown in numerous studies (Crowe and Higgins, 1997; Higgins, 1997; Shah et al., 1998; Liberman et al., 1999; Freitas and Higgins, 2002; Chernev, 2004; Avnet and Higgins, 2006), promotion focus individuals use approach and eagerness means to pursue their goals, tend to make changes and to take risks, try to achieve gains, and are prone to action; whereas prevention focus individuals use avoidance and vigilance means to pursue their goals, tend to maintain stability and to keep the status quo, try to avoid losses, and tend to caution and inaction. The inaction preference can be so profound that prevention-focused individuals might choose the status quo even if it is not the profitable one (Chernev, 2004).

According to regulatory focus theory, when the individuals' regulatory focus matches their goal pursuit means, they experience regulatory fit, which subsequently enhances their belief in what they are doing and the significance of their decisions (Higgins, 2000, 2005, 2006). Promotion focus fits goal pursuit means, such as eagerness and approach strategies (e.g., taking risks), whereas prevention focus fits goal pursuit means, such as vigilance and avoidance strategies (e.g., avoid risks). Under regulatory fit, people will judge a decision they made as more “right,” value it more, and feel more engaged to their decision, than under non-fit condition (Camacho et al., 2003; Higgins, 2005). For example, Camacho et al. (2003) asked subjects to imagine themselves having a conflict with another person and to evaluate the other person's strategy of resolving the conflict. Promotion-focused subjects evaluated eager strategies (e.g., encouraging you to succeed) as more right than prevention-focused subjects, whereas prevention-focused subjects evaluated vigilant strategies (e.g., removing anything that might cause trouble) as more right than promotion-focused subjects. Based on the regulatory fit principle, it can be argued that in situations of regulatory fit, because people feel more right about what they are doing, there will be less regret.

Although regret is a central emotion, the influence of regulatory fit on regret is still in the first stages of investigation. Kwak and Park (2008) showed that fear from anticipated regret increases the “sunk cost” effect (continue investing in a hopeless situation) under regulatory fit condition. However, our research focuses on the influence of regulatory fit on experienced regret in post-choice evaluation. Although conceptual models have suggested including regulatory fit as an integral part of the regret process, little has been conducted in this research area (Roese et al., 2007; Das and Kerr, 2010). We have found only one study that examined the effect of regulatory focus on regret (Church and Iyer, 2012), however, this study was not designed to test the different level of regret following action vs. inaction. The authors found (in contrast to their prediction) that people with higher promotion focus tended to regret their actions more than people higher on prevention focus. However, this was not compared to the level of regret following inaction. Thus, we cannot infer from these findings about our research questions.

In the present study we hypothesize that regulatory fit leads to less regret than regulatory non-fit. Specifically, a decision of action will fit individuals in promotion focus, and a decision of inaction will fit individuals in prevention focus. In other words, we argue that inaction will be considered as more normal behavior under prevention focus than under promotion focus, whereas action decisions will be viewed as more normal under promotion focus. Consequently, in line with norm theory (Kahneman and Miller, 1986), we hypothesize that the phenomenon of attaching more regret to action that fails than to inaction that fails, will be lower under promotion focus and higher under prevention focus. Thus, our hypotheses are:

***Hypothesis 1***: *When two decisions resulted in failure, one is an action decision and another is an inaction decision, individuals in promotion focus will be more likely to attribute the lower regret to the action decision than individuals in prevention focus*.
***Hypothesis 2***: *Individuals in promotion focus will attribute **less** regret than individuals in prevention focus to an **action** decision that resulted in failure*.
***Hypothesis 3***: *Individuals in promotion focus will attribute **more** regret than individuals in prevention focus to an **inaction** decision that resulted in failure*.

Hypothesis 1 relates to a binary question of regret (i.e., “Who feels more regret, the person who chose action or the person who chose inaction?”). Due to the binary nature of the question, this hypothesis is essentially identical to the symmetric hypothesis (“When two decisions resulted in failure, one is an action decision and another is an inaction decision, individuals in prevention focus will be more likely to attribute the lower regret to the inaction decision than individuals in promotion focus”) and therefore the results, which are presented according to Hypothesis 1, can also be interpreted as addressing this symmetric hypothesis.

Hypotheses 2 and 3 relate to two continuous variables of regret (i.e., level of regret following action and level of regret following inaction). Additionally, since regulatory focus is an individual tendency but could also be temporarily induced, all hypotheses will be tested by both individual and induced regulatory focus.

Along the lines of Zeelenberg et al. (2002), we hypothesize that a negative prior outcome makes action (a change in the status quo) more normal than absent such a negative prior outcome. In other words, a negative prior outcome creates a trigger for a change, and such a trigger increases the normality of choosing action and reduces the normality of choosing inaction, leading to reduced regret from failed action and increased regret from failed inaction. Moreover, we believe that not only a prior negative outcome but also a change in the environment may create a trigger for change, increasing the normality of action and reducing the normality of inaction, and therefore reducing the regret from action decisions compared to inaction decisions. For example, a trigger for change could stem from changing the targeted production level in one's work environment, which implies that a change in one's work strategy might be needed. To sum, we hypothesize:

***Hypothesis 4***: *A trigger for change (caused by prior negative outcome or by a change in the environment) will* ***reduce*** *regret after failed* ***action*** *and* ***increase*** *regret after failed* ***inaction***.

Several demographic variables might affect the tendency to regret action or inaction. People's level of religiosity may affect their tendency to favor action vs. inaction in different life situations. For example the Orthodox in Israel are known for their aversion of changes and by their clinging to the status quo, holding the motto of “to any proposal for change say ‘no”’ (Lehmann and Siebzehner, 2009). Therefore, they might show more regret following action. Other demographic variables, such as gender, age, and income level could also affect the level of regret and therefore were taken into account in our analysis.

## Methods

To test our hypotheses, we conducted an online experiment that included six scenarios in which regulatory focus (prevention vs. promotion) was induced, and regret level was measured after scenarios of failed action decisions vs. failed inaction decisions. In addition, chronic individual regulatory focus as well as demographic and personal variables were measured. The study received an approval from the Human Subjects Research Committee of the University.

### Sample

A total of 330 Israeli subjects were recruited voluntarily through a polling service company and were paid in exchange for their participation. One hundred and seventy three (52.4%) were female, age range was between 25 to 60 years old, and the mean age was *M* = 39 (*SD* = 10.14). Income level ranged between much below average (*n* = 41; 12.4%), below average (*n* = 69; 20.9%), average (*n* = 128; 38.7%), above average (*n* = 66; 20%), and much above average (*n* = 21; 6.3%), with 5 missing values. In terms of religiosity level, 171 (51.8%) were secular, 76 (23%) traditionalists, 45 (13.6%) orthodox, and 38 (11.5%) ultra-orthodox.

### Procedure

The study consisted of three parts. In the first part participants filled a consent form and then filled the chronic regulatory focus measure. In the second part the subjects were divided randomly into three treatment groups: Induced promotion focus (*n* = 94), induced prevention focus (*n* = 116), and a control group (*n* = 120). The manipulation included a word-completion task, in which subjects were asked to complete missing words in a text, using specific words that were provided in a list. The induced promotion focus manipulation used a list of promotion words (e.g., gain, aspirations, success), whereas the induced prevention focus manipulation used a list of prevention words (e.g., loss, obligations, failure). In the control condition no task was given. In order to check the manipulation the participants were asked to rate 8 behavior tendencies related to either a promotion focus (e.g., eager) or a prevention focus (e.g., vigilant), on a 10-point scale.

The third part of the experiment involved six scenarios. Each scenario presents an uncertain situation with two possible decisions: to retain the status quo (inaction) or to make a change (action). One decision maker in the scenario chooses action and the other chooses inaction, and both fail. The first two scenarios replicated those used in previous studies (Kahneman and Tversky, 1982; Zeelenberg et al., 2002), and the sixth scenario is somewhat similar to that in Gilovich and Medvec (1995). The additional new scenarios were developed to examine the robustness of the results to different contexts and situations, keeping the same structure of failed action vs. inaction. Three of the scenarios (2, 5, 6) contained a trigger for change, either a prior negative outcome or a change in the environment, while the other three scenarios did not contain any signal for the need of change (1, 3, 4). The scenarios were always presented in the same order, from 1 to 6.

After each scenario the subjects were asked to indicate who feels more regret (the one who acted or the one who did not act). This was the question used in Kahneman and Tversky (1982) and in Zeelenberg et al. (2002). Thus, in order to precisely replicate the original studies, we did not add any other questions that may affect the answers to the original question. In the four additional scenarios, however, we added two questions that asked the subjects to estimate the regret level of each decision maker on a 0–100 scale. We assumed that when participants are asked to estimate the regret level of a person in a hypothetical scenario they will use their own experience and personality to make their estimation. The six scenarios appear in the Appendix.

### Measures

*Individual Regulatory Focus* was measured with two measures: one is the scale of Lockwood et al. (2002), which is the most common scale for measuring regulatory focus, and the other is the Outcome-Based Measure (OBM; Schödl et al., 2013), a recently developed scale for regulatory focus.

1. *The Lockwood's Regulatory Focus Scale* consists of 18 items with a 9-point Likert scale. An example of a prevention item is “In general, I am focused on preventing negative events in my life,” and an example of a promotion item is “In general, I am focused on achieving positive outcomes in my life.” To calculate the total regulatory focus measure, an average score for each regulatory focus was calculated, then the gap between the promotion focus score to the prevention focus score was calculated (deducting the prevention score from the promotion score). The higher the score, the higher is the tendency toward promotion focus.
2. *The OBM Scale of Regulatory Focus* consists of 11-paired items describing cognitive, emotional, and strategic outcomes of regulatory focus based on Higgins (1997, 1998) theory. Each pair has two endings, one of promotion and one of prevention. A sample pair of items for a cognitive outcome is: “In general, I pay attention to: (a) negative information, (b) positive information.” A sample pair of items for an emotional outcome is: “When I complete a task successfully: (a) I feel relief, (b) I feel joy.” A sample pair of items for a strategic outcome is: “In general, I am: (a) enthusiastic, (b) cautious.” Both endings of each item are rated on a 0–10 scale (0 = not at all true about me, 10 = very true about me). Reliabilities were α = 0.83 for promotion and α = 0.82 for prevention. A score for each regulatory focus was calculated by the sum of the answers to the relevant endings (prevention or promotion). The total regulatory focus measure is then obtained by deducting the prevention focus score from the promotion focus score. The higher the score, the higher is the tendency toward promotion focus.

Demographic variables age, gender, income, and religiosity level, were provided by the polling service company. Income was measured on an ordinal five-point scale (much below average, below average, average, above average, much above average). Religiosity level contained four categories that represent four main Israeli sectors: secular, traditionalist, orthodox, and ultra-orthodox. We recoded religiosity into a dichotomous variable with secular and traditionalist coded as “0” (non-orthodox), and orthodox and ultra-orthodox coded as “1.”

## Results

### Induced regulatory focus manipulation

We first conducted a manipulation check for the regulatory focus manipulation. A set of eight Independent-Sample *t*-tests showed no differences between promotion and prevention conditions in terms of the behavior tendencies evoked by the word-completion task (*t*-tests ranged between −0.11 < *t* < 1.61; and significance levels.12 < *p* < 0.91). None of the eight behavior tendencies revealed significant difference between the two regulatory focus manipulations. As a result, the induced regulatory focus was not used in further analyses, but in order to control the potential effect of the manipulation, we added to the regressions two dummy variables for the promotion and prevention treatments (denoted by Promotion_Tr and Prevention_Tr in the regressions), where the control treatment with no word-completion task is the benchmark. Thus, further analyses tested hypotheses 1–3 only with regards to the individual measures of regulatory focus and not regarding the induced regulatory focus.

### Who feels more regret?

Next, we considered the first question in each scenario, asking which of the two decision makers feel more regret, the person who acted or the person who did not act (the dichotomous measure of regret). For each subject we only have a binary response about who felt more regret, but aggregating over all the subjects we can get the proportion of subjects that attributed more regret to action or to inaction. Table 1 presents these proportions and the test of whether the underlying probability is different from 0.5 (using the binomial distribution).

**Table 1:** **Who feels more regret–the person choosing action or inaction**?

**Scenario**	**Regret following action is higher**			**Regret following inaction is higher**		
	***N***	**Frequency**	**Percent (%)**	**Frequency**	**Percent (%)**	***p*-value (2-tailed)**
1	330	246	74.5	84	25.5	0.000
2	330	148	44.8	182	55.2	0.069
3	307	211	68.7	96	31.3	0.000
4	297	212	71.3	85	28.7	0.000
5	314	157	50	157	50	1.000
6	299	169	56.5	130	43.5	0.028

*The right column presents the 2-tailed p-value of the test (using the binomial distribution) of whether the probability of a subject attributing more regret to action (or inaction) is different from 0.5*.

The results show that in scenario 5 the proportions are exactly 50–50% and in scenario 2 more people attribute greater regret to inaction (55.2 vs. 44.8%), but the difference is not statistically significant. In the other four scenarios a higher regret was attributed more often to the person who acted than to the person who did not act, and the difference is statistically significant at the 5%-level (using a 2-tailed binomial test). Table 1 also shows that in three scenarios (2, 5, and 6) there were less than 57% who attributed more regret to action (than inaction). In the other three scenarios (1, 3, 4) more than 68% attributed more regret to action. The difference between these two groups of scenarios will be discussed later.

To test how regulatory focus is related to regret following action vs. inaction, we conducted two sets of six logistic regressions (one for each scenario) on the dichotomous measure of regret, namely, which person regret more, the one who acted (coded 1) or the one who did not act (coded 0). The first set of regressions included the following predictors: Demographics (age, gender, religiosity, and income), two dummy variables of the manipulation treatment of regulatory focus, and the individual regulatory focus measure of Lockwood. The second set of regressions was similar but used the OBM scale instead of Lockwood as the measure of individual regulatory focus. The results of the 12 logistic regressions, summarized in Table 2, show some support for Hypothesis 1. Specifically, Table 2 demonstrates that in scenarios 2 and 5 (with both measures of regulatory focus), and scenario 6 (only with the OBM scale), individual regulatory focus had a significant effect in the predicted direction, namely, the higher the promotion focus the lesser the probability of attributing more regret to the person who acted (compared with the one who did not act).

**Table 2:** **Logistic regressions: does action produce more regret than inaction**?

**Scenario**	**Variables**	**Lockwood's scale**			**OBM Scale**		
		**Coef**.	**Std. Err**.	***P***	**Coef**.	**Std. Err**.	***P***
1	Age	−0.010	0.0131	0.405	−0.013	0.013	0.321
	Female	−0.003	0.263	0.990	−0.009	0.265	0.972
	Religiosity	0.614	0.339	0.070	0.643	0.341	0.060
	Income	0.050	0.119	0.673	0.068	0.119	0.568
	Promotion_Tr	−0.333	0.310	0.282	−0.340	0.310	0.273
	Prevention_Tr	0.087	0.316	0.783	0.100	0.317	0.752
	Lockwood's promotion	0.075	0.072	0.301			
	OBM promotion				−0.012	0.089	0.890
2	Age	−0.029	0.012	0.018	−0.025	0.012	0.043
	Female	−0.021	0.232	0.928	−0.071	0.231	0.759
	**Religiosity**	**0.707**	**0.278**	**0.011**	**0.672**	**0.270**	**0.013**
	Income	−0.086	0.110	0.430	−0.113	0.110	0.306
	Promotion_Tr	−0.355	0.294	0.228	−0.299	0.294	0.310
	Prevention_Tr	−0.054	0.267	0.839	0.002	0.269	0.992
	**Lockwood's promotion**	**−0.178**	**0.062**	**0.004**			
	**OBM promotion**				**−0.191**	**0.080**	**0.016**
3	Age	0.016	0.012	0.204	0.017	0.012	0.168
	Female	0.197	0.253	0.435	0.152	0.257	0.554
	Religiosity	0.564	0.308	0.067	0.581	0.312	0.062
	Income	−0.060	0.108	0.577	−0.062	0.105	0.553
	Promotion_Tr	0.550	0.319	0.085	0.579	0.321	0.072
	Prevention_Tr	0.331	0.293	0.259	0.383	0.295	0.194
	Lockwood's promotion	−0.067	0.074	0.362			
	OBM promotion				−0.134	0.086	0.122
4	Age	0.018	0.014	0.196	0.018	0.014	0.196
	Female	−0.212	0.266	0.427	−0.216	0.269	0.422
	Religiosity	0.340	0.306	0.267	0.346	0.309	0.262
	Income	−0.042	0.117	0.720	−0.037	0.114	0.745
	Promotion_Tr	0.128	0.332	0.698	0.129	0.330	0.697
	Prevention_Tr	−0.116	0.304	0.702	−0.111	0.305	0.715
	Lockwood's promotion	0.010	0.073	0.894			
	OBM promotion				−0.007	0.081	0.927
5	Age	−0.009	0.012	0.467	−0.004	0.012	0.714
	Female	0.017	0.241	0.944	−0.029	0.243	0.903
	**Religiosity**	**0.787**	**0.290**	**0.007**	**0.747**	**0.286**	**0.009**
	Income	−0.101	0.111	0.365	−0.132	0.106	0.213
	Promotion_Tr	−0.552	0.298	0.065	−0.499	0.296	0.092
	Prevention_Tr	−0.141	0.279	0.612	−0.095	0.277	0.731
	**Lockwood's promotion**	**−0.184**	**0.063**	**0.004**			
	**OBM promotion**				**−0.174**	**0.080**	**0.030**
6	Age	−0.004	0.012	0.728	−0.003	0.012	0.796
	Female	0.373	0.240	0.120	0.294	0.243	0.226
	Religiosity	0.132	0.284	0.641	0.171	0.295	0.562
	Income	0.188	0.099	0.058	0.203	0.101	0.044
	Promotion_Tr	−0.247	0.299	0.409	−0.198	0.302	0.511
	Prevention_Tr	−0.270	0.282	0.339	−0.166	0.289	0.566
	Lockwood's promotion	−0.088	0.065	0.178			
	**OBM promotion**				**−0.252**	**0.084**	**0.003**
1–6 total	Age	−0.004	0.005	0.480	−0.002	0.005	0.668
	Female	0.059	0.110	0.592	0.018	0.111	0.868
	**Religiosity**	**0.494**	**0.138**	**0.000**	**0.497**	**0.140**	**0.000**
	Income	−0.013	0.0474	0.778	−0.019	0.048	0.682
	Promotion_Tr	−0.147	0.138	0.288	−0.118	0.138	0.391
	Prevention_Tr	−0.039	0.128	0.763	0.006	0.127	0.964
	**Lockwood's promotion**	**−0.074**	**0.031**	**0.017**			
	**OBM promotion**				**−0.124**	**0.037**	**0.001**

*The dependent variable is ActMoreRegret, a dummy variable that equals one if the subject thinks that the person who acted feels more regret than the one who did not act. The table reports the robust standard errors. The last regressions, on the combined data of scenarios 1–6, are clustered by subject ID*.

*Significant effects are bold*.

To be able to analyze the six scenarios together and derive more general conclusions, we created a database that aggregates the scenarios but records the unique subject ID in each observation. We then ran regressions on the combined data of scenarios 1–6 (clustered by subject ID), which are reported at the bottom of Table 2. These two regressions revealed that individual regulatory focus was statistically significant in the predicted direction (*p* = 0.017 using Lockwood's scale, *p* = 0.001 using the OBM scale). That is, the higher the promotion level, the less likely is the subject to attribute greater regret to the action decision (vs. inaction).

To sum, the effect of regulatory focus on the likelihood of attributing lower regret to the action decision (compared with the inaction decision) was obtained in three out of six scenarios when they are considered separately, and in the total measure of regret across all six scenarios. In addition, except for scenario 6, these effects were consistent across two different measures of regulatory focus. Thus, our results partially support hypothesis 1.

In addition to the effect of regulatory focus, subjects' religiosity level also had a significant effect on attributed regret. Specifically, in scenarios 2 and 5 and in the aggregated scenarios 1–6 (see Table 2) religiosity was positively and significantly related to the probability of attributing more regret to the person who acted. In scenarios 1 and 3 the effect of religiosity was positive and marginally significant (p-levels ranged between 0.06 and 0.07). All these mentioned effects of religiosity were consistently found across the two sets of regressions with both scales of regulatory focus. The positive effect of religiosity indicates that orthodox people are more likely than non-orthodox people to attribute more regret to action (compared to inaction).

### Regret levels following action vs. inaction

In order to test Hypothesis 2 we conducted two sets of linear regressions on the continuous measure of regret following action, which was measured in scenarios 3–6. Two sets of four linear regressions (for each of the four scenarios 3–6) were conducted on the level of regret attributed to the person who acted in the scenario. The predictors in the logistic regressions were used also here. In addition, the level of regret attributed to the person who did not act (by the same subject in the same scenario) was also included as an independent variable, in order to control for regret following inaction when predicting regret following action.

Table 3 presents the regression results that show what affects the regret attributed to the action decision. Because the question about the level of regret after the action decision was introduced only in scenarios 3–6, the results do not include scenarios 1–2. In scenarios 4, 5 (with both measures of regulatory focus), and 6 (only with the OBM scale), individual regulatory focus was significant at the 5% level in the predicted direction, namely, the higher the promotion focus, the lower the regret level attributed to the action decision. This effect was obtained beyond the positive effect of the level of regret attributed to the inaction decision. In other words, despite the fact that the level of regret attributed to inaction was positively and significantly related to the level of regret attributed to the action decision, the unique effect of regulatory focus on regret attributed to action was significant, supporting Hypothesis 2. To get an overview of the general findings across all scenarios, we also ran two regressions on the combined data of scenarios 3–6 (clustered by subject ID), reported at the bottom of Table 3. In line with Hypothesis 2, individual regulatory focus measured by both Lockwood's scale and the OBM scale was significant, such that the higher the promotion focus, the lower the regret attributed to the action decision.

**Table 3:** **Linear regressions explaining regret following action**.

**Scenario**	**Variables**	**Lockwood's scale**			**OBM scale**		
		**Coef**.	**Std. Err**.	***P***	**Coef**.	**Std. Err**.	***P***
3	Regret for inaction	**0.191**	**0.067**	**0.005**	**0.194**	**0.068**	**0.005**
	Age	0.016	0.153	0.918	0.046	0.154	0.763
	Female	0.660	2.762	0.811	0.149	2.787	0.957
	Religiosity	1.592	3.120	0.610	1.448	3.070	0.638
	Income	−1.049	1.285	0.415	−1.215	1.339	0.365
	Promotion_Tr	−0.204	3.129	0.948	0.278	3.119	0.929
	Prevention_Tr	3.304	3.190	0.301	3.930	3.200	0.220
	Lockwood's promotion	−1.375	0.764	0.073			
	OBM promotion				−1.758	0.939	0.062
4	Regret for inaction	**0.181**	**0.065**	**0.006**	**0.184**	**0.065**	**0.005**
	Age	0.039	0.159	0.803	0.077	0.161	0.631
	Female	1.568	2.831	0.580	0.697	2.867	0.808
	Religiosity	0.437	3.291	0.894	0.355	3.274	0.914
	Income	0.946	1.309	0.470	0.785	1.401	0.576
	Promotion_Tr	−5.223	3.319	0.117	−4.501	3.281	0.171
	Prevention_Tr	−2.666	3.119	0.393	−1.620	3.111	0.603
	**Lockwood's promotion**	−**1.868**	**0.739**	**0.012**			
	**OBM promotion**				−**2.894**	**0.875**	**0.001**
5	Regret for inaction	**0.141**	**0.069**	**0.041**	**0.148**	**0.068**	**0.031**
	Age	0.170	0.137	0.215	0.220	0.138	0.112
	Female	1.027	2.819	0.716	0.119	2.863	0.967
	Religiosity	3.900	3.518	0.268	3.730	3.467	0.283
	Income	−0.953	1.330	0.474	−1.209	1.370	0.378
	Promotion_Tr	−4.840	3.339	0.148	−4.026	3.287	0.222
	Prevention_Tr	−4.649	3.324	0.163	−3.589	3.356	0.286
	**Lockwood's promotion**	−**2.260**	**0.796**	**0.005**			
	**OBM promotion**				−**3.062**	**0.988**	**0.002**
6	Regret for inaction	**0.227**	**0.072**	**0.002**	**0.235**	**0.070**	**0.001**
	Age	0.035	0.156	0.823	0.055	0.159	0.730
	Female	2.393	3.034	0.431	1.69	3.073	0.582
	Religiosity	2.227	3.563	0.532	2.370	3.491	0.498
	Income	0.359	1.333	0.788	0.318	1.381	0.818
	Promotion_Tr	−2.040	3.788	0.590	−1.480	3.761	0.694
	Prevention_Tr	−3.369	3.517	0.339	−2.471	3.494	0.480
	Lockwood's promotion	−1.133	0.836	0.176			
	**OBM promotion**				−**2.340**	**1.053**	**0.027**
3–6 total	Regret for inaction	−.053	0.054	0.320	−.045	0.054	0.407
	Age	0.050	0.130	0.703	0.090	0.131	0.492
	Female	2.085	2.344	0.374	1.335	2.362	0.572
	Religiosity	1.418	2.864	0.620	1.276	2.828	0.652
	Income	0.183	1.345	0.892	−0.028	1.414	0.984
	Promotion_Tr	−3.499	2.588	0.176	−2.827	2.530	0.264
	Prevention_Tr	−1.944	2.735	0.477	−1.061	2.759	0.701
	**Lockwood's promotion**	−**1.845**	**0.652**	**0.005**			
	**OBM promotion**				−**2.501**	**0.809**	**0.002**

*The dependent variable is the regret (on a 0–100 scale) following a failed action decision. The table reports the robust standard errors. The last regressions, on the combined data of scenarios 3–6, are random-effects GLS regressions clustered by subject ID*.

*Significant effects are bold*.

To sum, the effect of regulatory focus on the attribution of regret to an action decision was obtained in three out of four scenarios and in the total measure of regret across all four scenarios. In addition, these effects were consistent across two different measures of regulatory focus. Thus, our results support hypothesis 2.

In order to test Hypothesis 3, we ran two additional sets of regressions on the level of regret following *inaction*. The same predictors were used as in the previous regressions, but this time we controlled for the regret level following *action*, since we predicted the level of regret following inaction. The results of these linear regressions are shown in Table 4 and surprisingly do not support Hypothesis 3. Specifically, no effect of regulatory focus on the level of regret following inaction was revealed (except for one effect of the Lockwood's scale in scenario 6). As can be seen in Table 4 the regret level following action positively predicts the level of regret following inaction, but regulatory focus has no unique effect on regret following inaction. Thus, the results did not confirm Hypothesis 3.

**Table 4:** **Linear regressions explaining regret following inaction**.

**Scenario**	**Variables**	**Lockwood's scale**			**OBM scale**		
		**Coef**.	**Std. Err**.	***P***	**Coef**.	**Std. Err**.	***P***
3	Regret for action	**0.209**	**0.070**	**0.003**	**0.21244**	**0.0711**	**0.003**
	Age	−0.147	0.149	0.325	−0.1552	0.150	0.302
	Female	2.368	2.821	0.402	2.692	2.830	0.342
	Religiosity	−3.087	3.154	0.328	−3.155	3.138	0.315
	Income	1.665	1.247	0.183	1.670	1.231	0.176
	Promotion_Tr	−2.799	3.410	0.412	−3.037	3.409	0.374
	Prevention_Tr	−5.534	3.248	0.089	−5.956	3.212	0.065
	Lockwood's promotion	0.474	0.779	0.543			
	OBM promotion				1.073	1.035	0.301
4	Regret for action	**0.210**	**0.075**	**0.005**	**0.216**	**0.076**	**0.005**
	Age	0.132	0.161	0.413	0.1372	0.161	0.394
	Female	**6.566**	**2.978**	**0.028**	**6.698**	**2.982**	**0.025**
	Religiosity	1.489	3.389	0.661	1.333	3.381	0.694
	Income	0.259	1.299	0.842	0.177	1.297	0.891
	Promotion_Tr	2.055	3.569	0.565	2.026	3.551	0.569
	Prevention_Tr	2.105	3.429	0.540	1.916	3.443	0.578
	Lockwood's promotion	−0.102	0.833	0.902			
	OBM promotion				0.438	1.094	0.689
5	Regret for action	**0.151**	**0.072**	**0.037**	**0.159**	**0.071**	**0.027**
	Age	−0.138	0.158	0.381	−0.134	0.160	0.405
	Female	3.417	2.918	0.242	3.616	2.943	0.220
	Religiosity	−5.634	3.507	0.109	−5.854	3.467	0.092
	Income	1.845	1.308	0.159	1.755	1.309	0.181
	Promotion_Tr	−0.523	3.653	0.886	−0.575	3.636	0.875
	Prevention_Tr	3.353	3.356	0.318	3.103	3.370	0.358
	Lockwood's promotion	−0.098	0.824	0.905			
	OBM promotion				0.627	1.005	0.533
6	Regret for action	**0.216**	**0.068**	**0.002**	**0.230**	**0.068**	**0.001**
	Age	−0.132	0.154	0.391	−0.081	0.156	0.605
	Female	−1.683	2.983	0.573	−1.561	3.037	0.608
	Religiosity	−4.416	3.586	0.219	−5.222	3.617	0.150
	Income	1.985	1.298	0.127	1.497	1.303	0.251
	Promotion_Tr	−2.768	3.706	0.456	−2.527	3.779	0.504
	Prevention_Tr	0.493	3.479	0.887	0.265	3.530	0.940
	**Lockwood's promotion**	−**1.724**	**0.781**	**0.028**			
	OBM promotion				0.171	0.936	0.855
3–6 total	Regret for action	−0.022	0.059	0.702	−0.014	0.059	0.805
	Age	−0.061	0.121	0.611	−0.040	0.121	0.744
	Female	3.095	2.247	0.169	3.132	2.244	0.163
	Religiosity	−2.594	2.723	0.341	−2.937	2.706	0.278
	Income	1.475	1.254	0.240	1.272	1.261	0.313
	Promotion_Tr	−1.733	2.606	0.506	−1.612	2.598	0.535
	Prevention_Tr	−0.288	2.562	0.910	−0.375	2.587	0.885
	Lockwood's promotion	−0.751	0.601	0.212			
	OBM promotion				0.035	0.792	0.964

*The dependent variable is the regret (on a 0–100 scale) following a failed inaction decision. The table reports the robust standard errors. The last regressions, on the combined data of scenarios 3–6, are random-effects GLS regressions clustered by subject ID*.

*Significant effects are bold*.

### The effect of a trigger for change

We now turn to examine Hypothesis 4, according to which a trigger for change lowers the level of regret attributed to action. Scenarios 2, 5, and 6, included a trigger for change, whereas scenarios 1, 3, and 4 did not include any trigger for change. Scenario 2, which replicates a study of Zeelenberg et al. (2002), includes a negative prior outcome (losing the prior game), after which the coach has to decide whether to change the team. The prior loss creates a trigger to do something different, i.e., a trigger for change. Similarly, in Scenario 6, which deals with a decision of students to change or not their university, it is mentioned that the students are unhappy with their university, again creating a trigger for change. In scenario 5, which deals with two employees who have weekly manufacturing targets, it is mentioned that this week the target was higher than usual. This is not a prior negative outcome but it is an important change in the environment, which can be a trigger for change in the decision (which machine parameters to adopt). In contrast to those three scenarios, scenarios 1, 3, and 4, describe a decision of two people to change or not to change, without any additional information that could be a trigger for change. For example, scenario 1 (a replication of a scenario from Kahneman and Tversky, 1982) describes two people who decide to change/not change a stock, but no reason or additional information regarding the necessity of a change is given. Similarly, scenarios 3 and 4 present two decisions to change/not change a project (scenario 3), or a supplier (scenario 4), but no additional information is given for a prior negative outcome of the current project or supplier, or a significant change in the environment. Therefore, no apparent trigger for change is created in scenarios 1, 3, and 4.

Table 1 already demonstrates that something is different between the trigger for change (TFC) scenarios 2, 5, and 6, and the no-TFC scenarios 1, 3, and 4. In particular, the proportion of subjects who attribute greater regret to action than to inaction ranged between 68.7 and 74.5% in the no-TFC scenarios, but only 44.8–56.5% in the TFC scenarios. Considering the continuous variables of regret levels following action and inaction, we see again a remarkable difference between the TFC scenarios (now only scenarios 5 and 6 because no continuous regret levels were elicited for scenarios 1 and 2) and the no-TFC scenarios 3 and 4. More specifically, in the no-TFC scenarios (3 and 4), the regret from action was higher than regret from inaction and the difference was statistically significant (68.31 vs. 56.29, *p* = 0.0000 in Scenario 3; 69.33 vs. 55.94, *p* = 0.0000 in Scenario 4). However, in the TFC scenarios (5 and 6) the regret levels from action and inaction were very close and not statistically significant (61.38 vs. 58.35, *p* = 0.1027 in Scenario 5; 59.27 vs. 57.79, *p* = 0.4274 in Scenario 6). Overall, the level of regret after action was significantly higher when no trigger for change exists compared to the TFC scenarios (68.82 vs. 60.33, *p* = 0.0000). However, the regret from inaction was similar regardless of a trigger for change (56.11 vs. 58.07, *p* = 0.1747).

Hypothesis 4 was tested on the combined data of scenarios 1–6 (clustered by subject ID). We ran logistic regressions on the dichotomous measure of regret with the same independent variables as in the previous logistic regressions, but also adding a dummy variable for the trigger for change (coded “0” for no-TFC scenarios, and “1” for TFC scenarios). In addition, in order to test whether the effect of regulatory focus differs between TFC and no-TFC scenarios, we added the interaction between the trigger for change and the individual regulatory focus (TFC X promotion focus). Table 5 summarizes the results of the two regressions (one with Lockwood's promotion focus and one with the OBM promotion focus).

**Table 5:** **Logistic regressions explaining regret following action vs. inaction: adding the trigger for change**.

**Variables**	**Lockwood's scale**			**OBM scale**		
	**Coef**.	**Std. Err**.	***P***	**Coef**.	**Std. Err**.	***P***
Age	−0.004	0.006	0.489	−0.002	0.006	0.667
Female	0.062	0.116	0.592	0.020	0.117	0.864
**Religiosity**	**0.519**	**0.145**	**0.000**	**0.521**	**0.147**	**0.000**
Income	−0.011	0.051	0.832	−0.017	0.051	0.734
Promotion_Tr	−0.156	0.146	0.287	−0.125	0.145	0.390
Prevention_Tr	−0.043	0.135	0.751	0.005	0.134	0.971
**TFC**	−**0.710**	**0.135**	**0.000**	−**0.867**	**0.105**	**0.000**
Lockwood's promotion	−0.008	0.042	0.838			
**TFC X Lockwood's promotion**	−**0.125**	**0.051**	**0.014**			
OBM Promotion				−0.052	0.048	0.277
**TFC X OBM promotion**				−**0.148**	**0.065**	**0.022**

*The dependent variable is ActMoreRegret, a dummy variable that equals one if the subject thinks that the person who acted feels more regret than the one who did not act. TFC, Trigger for Change. The table reports the robust standard errors. The regressions are clustered by subject ID*.

*Significant effects are bold*.

As can be seen in Table 5, according to our prediction, the trigger for change had a significant negative effect on the probability of attributing more regret to action, meaning that when there is a trigger for change, less regret is attributed to action (compared to no trigger for change). This finding was consistent across the two measures of individual regulatory focus and further confirmed Hypothesis 4. In addition, while the main effect of regulatory focus was non-significant, the interaction between regulatory focus and the trigger for change was significant and negative. This significant interaction together with the lack of significant effect of the promotion focus variable itself, suggests that when asking subjects the binary question of who feels more regret, there is no significant effect of promotion focus in scenarios without a trigger for change, but there is a significant effect of promotion focus once a trigger for change is introduced. In particular, a trigger for change makes it less likely that the greater regret will be attributed to the person who chose action. These findings were consistent across the two measures of individual regulatory focus.

In addition, subjects' religiosity level also had a significant effect on attributed regret, indicating that orthodox people are more likely than non-orthodox people to attribute more regret to action (*p* = 0.000 for both measures of regulatory focus). This effect was consistent with the effects of religiosity that were found in the previous logistic regressions (see Table 2).

We also tested Hypothesis 4 on the two continuous measures of regret: regret following action (see Table 6) and regret following inaction (see Table 7). Two sets of linear regression models were conducted on the combined data of scenarios 3–6 (clustered by subject ID). The independent variables were the same as in the previous regression, except that we controlled for regret following inaction when predicting regret following action; and we controlled regret following action when predicting regret following inaction.

**Table 6:** **Linear regressions explaining regret following action: adding the trigger for change**.

**Variables**	**Lockwood's scale**			**OBM scale**		
	**Coef**.	**Std. Err**.	***P***	**Coef**.	**Std. Err**.	***P***
Regret for Inaction	−0.055	0.053	0.302	−0.045	0.053	0.392
Age	0.050	0.131	0.704	0.090	0.131	0.493
Female	2.088	2.346	0.373	1.338	2.363	0.571
Religiosity	1.415	2.868	0.622	1.274	2.831	0.653
Income	0.184	1.347	0.891	−0.027	1.415	0.985
Promotion_Tr	−3.501	2.591	0.177	−2.828	2.534	0.264
Prevention_Tr	−1.944	2.739	0.478	−1.061	2.763	0.701
**TFC**	−**7.794**	**1.434**	**0.000**	−**8.263**	**1.254**	**0.000**
**Lockwood's promotion**	−**1.645**	**0.693**	**0.018**			
TFC X Lockwood's promotion	−0.401	0.636	0.529			
**OBM promotion**				−**2.210**	**0.820**	**0.007**
TFC X OBM promotion				−0.581	0.751	0.440

*The dependent variable is the regret (on a 0–100 scale) following a failed action decision. TFC, Trigger for Change. The table reports the robust standard errors. The regressions are random-effects GLS regressions clustered by subject ID*.

*Significant effects are bold*.

**Table 7:** **Linear regressions explaining regret following inaction: adding the trigger for change**.

	**Lockwood's scale**			**OBM scale**		
**Variables**	**Coef**.	**Std. Err**.	***P***	**Coef**.	**Std. Err**.	***P***
Regret for action	−0.013	0.060	0.822	−0.004	0.061	0.947
Age	−0.062	0.120	0.607	−0.041	0.121	0.736
Female	3.077	2.237	0.169	3.119	2.231	0.162
Religiosity	−2.608	2.710	0.336	−2.952	2.690	0.272
Income	1.474	1.245	0.237	1.273	1.249	0.308
Promotion_Tr	−1.702	2.595	0.512	−1.582	2.585	0.540
Prevention_Tr	−0.271	2.550	0.915	−0.364	2.572	0.887
**TFC**	**3.557**	**1.736**	**0.040**	2.085	1.440	0.148
Lockwood's promotion	−0.264	0.725	0.715			
TFC X Lockwood's promotion	−0.941	0.758	0.214			
OBM promotion				0.128	0.969	0.895
TFC X OBM promotion				−0.131	0.879	0.881

*The dependent variable is the regret (on a 0–100 scale) following a failed inaction decision. TFC, Trigger for Change. The table reports the robust standard errors. The regressions are random-effects GLS regressions clustered by subject ID*.

*Significant effects are bold*.

As can be seen in Table 6, the trigger for change had a significant negative effect on regret following action, meaning that when there is a trigger for change, less regret is attributed to action. This finding further confirms Hypothesis 4. In addition, the effect of regulatory focus was significant such that the higher the promotion focus, the lower the regret following action (supporting Hypothesis 2 as in our earlier findings). The interaction between trigger for change and regulatory focus was non-significant. This pattern of results was consistent in both measures of individual regulatory focus.

Finally, as can be seen in Table 7 and in line with Hypothesis 4, the trigger for change had a positive effect on regret following inaction, meaning that when there is a signal that a change might be needed, there is more regret following inaction. However, this effect was weaker than the effect of TFC on regret from action (the coefficients of TFC on regret from action are −7.8 and −8.3 in the two regressions, compared to coefficients of +3.6 and +2.1 on regret from inaction). In addition, this effect was statistically significant for the regret from inaction only when the Lockwood's scale was used. When using the OBM scale this effect was not statistically significant, although it had a positive coefficient as predicted. The individual regulatory focus had no effect on regret following inaction, similar to the results in Table 4, and once again not consistent with Hypothesis 3. The interaction between individual regulatory focus and the trigger for change also had no effect on regret from inaction.

In sum, the data strongly support our prediction that the existence of a trigger for change decreases the level of regret following action, but only partially support our prediction that it increases the level of regret following inaction. In addition, the effect of regulatory focus was similar to our earlier findings, namely, promotion focus decreases regret following action (supporting Hypothesis 2), but does not increase regret following inaction (not supporting Hypothesis 3).

### Summary of results

Our results provide partial support for hypotheses 1, 2, and 4, but did not support hypothesis 3. When testing whether more regret is attributed to action decision or to inaction decision, we found that regulatory focus was significantly related to regret in three out of six scenarios (2, 5, and 6) and when the effect is calculated across all six scenarios. The direction of the effect indicates that the higher the promotion focus, the lower the probability of attributing more regret to action. Similarly, when testing the regret following action (where it was measured on a 0–100 scale, i.e., in scenarios 3–6), the same effect of regulatory focus was found. Specifically, regulatory focus was related to regret in three out of four scenarios (4, 5, and 6) and when the effect is calculated across all four scenarios, such that the higher the promotion focus, the lower the attributed regret following action. However, when testing regret following inaction, there was no effect of regulatory focus in any of the scenarios (except for scenario 6 in Lockwood's scale), and also not when calculating the total effect across all four scenarios. In addition, according to our prediction, we found that when the situation contains a trigger for change, less regret is attributed to action and more regret is attributed to inaction (although the effect of TFC on inaction was not always statistically significant and it was weaker than its effect on action). Finally, relatively high consistency was found in the results pattern between the two scales of regulatory focus. This consistency further strengthens the robustness of our findings.

## Discussion and conclusions

The present study examines the effect of regulatory focus on regret feelings following action vs. inaction decisions. The results indicate that individual differences in regulatory focus are related to the level of regret that emerges after making a decision that results in failure, and in particular, after making an action decision. The mechanism that explains the effect of regulatory focus on regret stems from the principle of regulatory fit. According to this principle, when the individual regulatory focus of decision makers fits their goal pursuit means or strategies, they feel more right about what they are doing (Higgins, 2000, 2005, 2006). Because an action decision fits more promotion focus, whereas an inaction decision fits more prevention focus (e.g., Chernev, 2004), we predicted that action will be less regrettable for promotion-focused individuals, whereas inaction will be less regrettable for prevention-focused individuals. Our results indeed show that promotion-focused individuals attribute less regret to action decisions than prevention-focused individuals. However, no difference was found between individuals with promotion and prevention foci with regard to regret from inaction decisions.

### Regulatory focus and regret

Our findings contribute to the regulatory focus research arena by expanding the role of individual regulatory focus to the domain of regret. So far numerous studies have found that regulatory focus affects people's decisions and choices (e.g., Aaker and Lee, 2001; Chernev, 2004; Lee and Aaker, 2004; Avnet and Higgins, 2006), strategies (e.g., Crowe and Higgins, 1997; Lockwood et al., 2002) and emotions (e.g., Higgins et al., 1997). However, as far as we know no study has investigated the effect of regulatory focus on post-choice regret. While previous research showed that people valued more decisions that were made under conditions of regulatory fit, than under non-fit (e.g., Higgins et al., 2003), the current research extends previous research by showing that under regulatory fit condition, people are also less likely to regret their decisions. Specifically, since action decision fits promotion focus orientation, an action decision is regretted less by promotion-focused individuals than by prevention-focused individuals. Understanding the impact of regulatory focus on regret from action vs. inaction could have implications for individuals' well-being and emotional regulation. For example, we can predict that prevention-focused individuals will be more sensitive to the negative effects of regret emerged by action decisions that failed; such negative effects could be reduced well-being, guilt or other negative feelings. On the other hand, our results do not suggest that the opposite effect is true for promotion-focused individuals, namely, inaction decisions that failed do not seem to harm promotion-focused individuals (compared to prevention ones). Thus, we suggest that prevention-focused individuals will be more sensitive to the harmful effect of regret following action decision, while promotion-focused individuals will be more resilient to such harmful effect. This notion is consistent with previous research suggesting that prevention-focused individuals might be more vulnerable to reduced well-being, whereas promotion focus is related to more resiliency (Van Dijk et al., 2013). Future studies are encouraged to further investigate the effect of regulatory focus on regret and regret consequences, such as reduced well-being, negative feelings and regret aversion.

### Action and inaction asymmetry

Our findings show asymmetry in the effect of regulatory focus on regret following action vs. inaction. This asymmetry has not been revealed by previous studies. When using a binary measure of regret (i.e., who regrets more: a person who acted or a person who did not act), we found that promotion focus decreased the probability of attributing more regret to action than to inaction. However, using the binary question we still do not know whether this effect results from promotion-focused individuals attributing less regret to action, more regret to inaction, or both. The use of additional two continuous measures of regret (i.e., regret following action and regret following inaction) revealed an asymmetric pattern between action and inaction. Specifically, promotion-focused individuals attribute less regret to action than prevention-focused individuals, but the two groups attribute similar regret levels to inaction decisions. This asymmetry between action and inaction implies that a decision not to act is the default or the norm, as suggested by the norm theory (Kahneman and Miller, 1986). This means that inaction or leaving things as they are, without making any change, is the first and basic option in a situation of choice. Taking action or changing the status quo, on the other hand, is a less trivial choice and it requires more intent and deliberate plan. Therefore, inaction decisions are similarly perceived by different individuals, and even among individuals who tend to use action strategies or a promotion focus–inaction is still an acceptable and normal option. Action decisions, on the other hand, are perceived as a desirable option only by individuals who are predisposed to action; since an action decision is beyond the default and it takes more effort and intent to choose it, such a decision will not fit all individuals. This extends the norm theory of Kahneman and Miller (1986), suggesting that individual differences (at least with respect to the regulatory focus) are more notable in regret following action than in regret following inaction. We encourage future studies to further test the asymmetric effect of individual regulatory focus as well as other individual tendencies on action vs. inaction decisions.

### Prior negative outcome and trigger for change

Zeelenberg et al. (2002) suggested that when there is information regarding a prior negative outcome, action decision becomes more normal and acceptable, and therefore is less regrettable, than when no such information exists. Our results support this idea that a prior negative outcome makes action more normal than otherwise and consequently reduces regret following action. However, we go beyond this and find that not only prior negative outcomes but also other situational cues that signal the need for change, such as changing a weekly target at work (scenario 5), reduce regret following action. We suggest that a trigger for change makes action more normal than without such a trigger, and therefore it reduces regret following action, in line with the norm theory of Kahneman and Miller (1986), which suggests that regret is greater when it follows less normal decisions. Our findings add to other studies that show particular situations in which action is the norm and therefore produces less regret, such as the decision of goalkeepers in penalty kicks to jump (Bar-Eli et al., 2007). However, although our results show that a trigger for change reduces the level of regret attributed to action, it is not reversing the regret attribution pattern. In the three scenarios that contained a trigger for change (2, 5 and 6), only in scenario 2 a reversed pattern was evident (i.e., inaction was perceived as more regrettable than action). However, even in scenario 2, where the percent of attributing more regret to action is only 44.8% (the lowest among the scenarios), it is not statistically significant different from 50%.

### Practical implications

An implementation of our results to decision making situations in both individual and organizational contexts would be to select promotion-focused individuals for decision making assignments in which actions must be made. Since there is less regret following action among promotion-focused individuals, it is more likely that such individuals will have less regret aversion and will be more willing to take action when it is needed. Examples of contexts in which action decisions are mostly preferred would be Hi-Tech industries, or organizations who operate in a dynamic and turbulent environments that require frequent changes in technology, products, human resources, and so on. Another context that requires action decisions would be an entrepreneurial environment, in which individuals must be creative and innovative, discover opportunities, and develop new products. We are not suggesting that only promotion-focused individuals are required to make decisions in such environments and contexts, but in comparison to stable environments, high doses of promotion-focused individuals would be desirable. In contrast, in stable and less dynamic environments, changes and action decisions are required less frequently, and therefore the advantage of promotion-focused individuals is less significant. Yet, as our results show, inaction decisions are generally more preferred and less regretted by all individuals, regardless of their regulatory focus. Therefore, in steady environments, we suggest that both prevention—and promotion-focused individuals will tend to prefer inaction decisions. However, this idea needs further examination in both lab and field studies.

Another practical implication for effective decision making in organizations stems from our findings regarding the effect of a “trigger for change.” In order to encourage action decisions (in contexts that require changes), a useful suggestion would be to provide such triggers for change. For example, a manager who emphasizes to the employees the differences between the current situation and the previous one creates more triggers for change than a manager who emphasizes the similarities between the situations or who does not emphasize anything. As another example, consider two universities in which the Dean asks the faculty to update their courses and propose beneficial changes to the program. In the first university the Dean emphasizes that due to increased competition from colleges there is a reduced demand for the program. In the second university, although the situation is similar, the Dean just asks to try to improve the program as much as possible, or may be even emphasizes the similarities (e.g., that after the proposed changes, courses should still be semester-based, and the BA should still take 4 years). The first Dean, who emphasizes the changes in the environment, creates a trigger for change, and therefore is likely to encourage a more proactive and innovative mindset, more changes, and more needed action than the second Dean who did not create a trigger for change. According to our findings, triggers for change reduce the level of regret from action decisions, and thus increase the tendency to adopt action decisions.

### Research limitations and future research

One limitation of our study is that the regulatory focus manipulation did not produce the expected effect. The current manipulation was chosen because we observed in other studies that Israeli subjects do not react as expected to the more common manipulations for regulatory focus (Higgins et al., 2001; Freitas and Higgins, 2002), i.e., these manipulations did not create promotion and prevention foci in Israeli samples. One possible reason is different interpretation of Israelis (compared to American subjects) of the terms used in Higgins' manipulations, namely oughts, duties, and obligations vs. ideals, dreams and aspirations. The manipulation that we have used is based on similar technique used by Lockwood et al. (2002) and it was recently tested by Schödl and Van Dijk (2014). Although the manipulation was independent of the individual measure of regulatory focus (because it was randomly allocated to subjects and because it was carried out after measuring individual regulatory focus), as another precaution we controlled for the potential effect of the manipulation by adding it as an independent variable in the regressions.

Another limitation of our study is that we tested the effect of regulatory focus on regret with hypothetical scenarios rather than creating true regret in the individual. However, creating real regret in the individual is very difficult. One needs to have the subject make a decision, then to make sure the decision results in failure so that a potential regret may arise. Even then, if the individuals do not attribute the failure to a significant mistake they made, they might not feel regret. For example, if one guesses the numbers in a lottery and then does not win, he probably does not feel strong regret, because there was no way in which he could know the winning numbers. So running an experiment in which subjects make decisions and the experimenter informs them that they made a mistake and they lose, will not necessarily create regret. Furthermore, even if one can design an experiment that creates real regret in the lab, it is likely to be regret about losing small amounts of money in an artificial setting. On the other hand, with the scenarios we were able to describe situations that involve more significant regret than losing a few dollars, and with a greater diversity of situations. By using six different hypothetical scenarios in different contexts, three different measures for regret, and two different measures for individual regulatory focus, we further increase the robustness, validity and the richness of the results. Although the above arguments explain our choice of hypothetical scenarios, it is a worthwhile direction for future research, albeit not an easy one, to think about lab experiments with real consequences that induce regret and use them to analyze how personality differences in general and regulatory focus in particular affect regret. Such studies may be interesting complements to our results.

Future studies should explore the impact of one's religiosity level on regret following action vs. inaction. Our findings show that orthodox people tend to attribute more regret than non-orthodox to a person who made an action decision. One explanation could be that orthodox people are more conservative and oriented to keep the status quo and avoid changes and risks. However, this finding is found only when using the dichotomous measure of regret and was not replicated with other measures of regret. Therefore, more research is needed in order to verify this effect.

Further research can be useful in order to verify our findings about the influence of regulatory focus on regret and confirm it in diverse situations, with different samples of subjects. We suggest to further explore the asymmetric effect of regulatory focus on action vs. inaction. An interesting direction would be to examine whether inaction is a type of decision that is perceived as the norm by most individuals, regardless of their personality, whereas an action decision is perceived differently according to the individual tendency, because it is considered as a less normal strategy. Another direction could be to present to the subjects various scenarios in different orders and analyze whether the order makes a difference. Additionally, the trigger for change should be tested in future studies in order to clarify and identify what types of information are perceived as a trigger for change, and consequently weaken the general tendency to regret more action than inaction decisions. Finally, the interaction effect that was found between the trigger for change and regulatory focus calls for future research to explore whether (and in what conditions) a trigger for change, which signals deviation from the norm, increases the impact of individual differences on regret feeling.

## Author contributions

AI: Experimental and study design, Analysis of the results, Writing the article. DV: Experimental and study design, Analysis of the results, Writing the article. OA: Experimental and study design, Analysis of the results, Writing the article.

### Conflict of interest statement

The authors declare that the research was conducted in the absence of any commercial or financial relationships that could be construed as a potential conflict of interest.

## Appendix: the scenarios used in the experiment

### Scenario 1: stock investment (Kahneman and Tversky, 1982)

Paul owns shares in Company A. During the past year he considered switching to stock in company B, but he decided against it. He now finds out that he would have been better off by $1200 if he had switched to the stock of Company B. George owned shares in Company B. During the past year he switched to stock in Company A. He now finds out that he would have been better off by $1200 if he had kept his stock in Company B. Who feels more regret?

### Scenario 2: soccer teams (Zeelenberg et al., 2002)

Jacob and Noah are both coaches of a soccer team. Jacob is the coach of team A, and Noah is the coach of team B. Both coaches lost the prior game with a score of 4–0. This Sunday Jacob decides to do something: He fields three new players. Noah decides not to change his team. This time both teams lose with 3–0. Who feels more regret, coach Jacob or coach Noah?

### Scenario 3: project management

Shirley and Rene are both project managers in a global company. As part of their jobs they decide with which projects to continue and which to terminate every quarter based on performance. At the beginning of the year, both of them were required to make a decision regarding projects that started earlier. Shirley decided to terminate project A and switch it with project B. Rene on the other hand decided to continue with project C that she started earlier. At the end of the year it turned out that both projects B and C failed, produced losses, and it was decided to terminate them.

1. Who feels more regret, Shirley or Rene?
2. a. What is the level of regret that Shirley feels on a scale of 0–100 (0 - no regret at all, 100 - very high level of regret)?
       b. What is the level of regret that Rene feels on a scale of 0–100 (0 - no regret at all, 100 - very high level of regret)?

### Scenario 4: supplier choice

Emma and Mia both work as purchasing managers in a big pharmaceutical company. As part of their jobs they decide with which raw materials suppliers to work. The company has been purchasing a variety of raw materials for the past five years from supplier A. Emma needed raw material X and received for it offers from both supplier A and supplier B, who is a supplier that has not yet been working with the company. Mia needed raw material Y and received for it offers from both supplier A and supplier C, who is a supplier that has not yet been working with the company. Emma decided to purchase the raw material X from the new supplier B. Mia decided to purchase the raw material Y from the old supplier A. After some time it was discovered that both new raw materials X and Y, from both suppliers B and A respectively, were of low quality and caused the company losses.

1. Who feels more regret, Emma or Mia?
2. a. What is the level of regret that Emma feels on a scale of 0–100 (0 - no regret at all, 100 - very high level of regret)?
       b. What is the level of regret that Mia feels on a scale of 0–100 (0 - no regret at all, 100 - very high level of regret)?

### Scenario 5: machine parameters

Michael and Daniel are both machine operators in a company that manufactures plastic products. Every week each of them receives his weekly target and has to make sure that the machine under his responsibility will produce this target. This week the target was higher than usual for both of them and therefore Michael and Daniel pondered what to do. Michael decided to change the machine parameters. Daniel decided to stay with the regular parameters. At the end of the week both Michael and Daniel did not succeed to reach the weekly target.

1. Who feels more regret, Michael or Daniel?
2. a. What is the level of regret that Michael feels on a scale of 0–100 (0 - no regret at all, 100 - very high level of regret)?
       b. What is the level of regret that Daniel feels on a scale of 0–100 (0 - no regret at all, 100 - very high level of regret)?

### Scenario 6: academic studies (based in part on Gilovich and Medvec, 1995)

Roy and Alex both studied for a Bachelor's degree in management and decided to continue to a Master's degree in business administration in the same university. After a short period in the degree both Roy and Alex felt that the degree is not contributing to them and the general feeling was that the attitude towards them is unpleasant and they do not enjoy the degree. Roy and Alex each considered whether to quit the university for a similar track in another university. Roy decided to stay and Alex decided to move to a different university. After half a year, they met and updated each other. They found that both of them are still unsatisfied with the degree they study.

1. Who feels more regret, Roy or Alex?
2. a. What is the level of regret that Roy feels on a scale of 0–100 (0 - no regret at all, 100 - very high level of regret)?
       b. What is the level of regret that Alex feels on a scale of 0–100 (0 - no regret at all, 100 - very high level of regret)?
